# The Effects of Cold Stress on Photosynthesis in *Hibiscus* Plants

**DOI:** 10.1371/journal.pone.0137472

**Published:** 2015-09-11

**Authors:** Miriam Paredes, María José Quiles

**Affiliations:** Departamento de Biología Vegetal, Facultad de Biología, Universidad de Murcia, Murcia, Spain; ISA, PORTUGAL

## Abstract

The present work studies the effects of cold on photosynthesis, as well as the involvement in the chilling stress of chlororespiratory enzymes and ferredoxin-mediated cyclic electron flow, in illuminated plants of *Hibiscus rosa-sinensis*. Plants were sensitive to cold stress, as indicated by a reduction in the photochemistry efficiency of PSII and in the capacity for electron transport. However, the susceptibility of leaves to cold may be modified by root temperature. When the stem, but not roots, was chilled, the quantum yield of PSII and the relative electron transport rates were much lower than when the whole plant, root and stem, was chilled at 10°C. Additionally, when the whole plant was cooled, both the activity of electron donation by NADPH and ferredoxin to plastoquinone and the amount of PGR5 polypeptide, an essential component of the cyclic electron flow around PSI, increased, suggesting that in these conditions cyclic electron flow helps protect photosystems. However, when the stem, but not the root, was cooled cyclic electron flow did not increase and PSII was damaged as a result of insufficient dissipation of the excess light energy. In contrast, the chlororespiratory enzymes (NDH complex and PTOX) remained similar to control when the whole plant was cooled, but increased when only the stem was cooled, suggesting the involvement of chlororespiration in the response to chilling stress when other pathways, such as cyclic electron flow around PSI, are insufficient to protect PSII.

## Introduction

Plants are frequently exposed to environmental stress, both in natural and horticultural conditions, and as a result growth may be affected. Understanding the physiological processes that underlie stress injury and the tolerance mechanisms of plants to environmental stress is of immense importance for both horticulture and the environment. Tolerance to stress results from integrated events occurring at all organization levels, from the anatomical and morphological to the cellular, biochemical and molecular levels. At the biochemical level, plants alter their metabolism in various ways to accommodate environmental stress, photosynthesis being one of these ways.

*Hibiscus rosa-sinensis* is a flowering plant species widely grown as an ornamental throughout the tropics, subtropics and also in temperate regions. Many plant species of tropical and subtropical origin manifest physiological dysfunctions when they are exposed to low, but non-freezing, temperatures of about 10–12°C [1,2]. Chilling injury under light has been attributed to the selective inhibition of the photosystems [3], PSII being the more sensitive to stress under light conditions, while PSI is more stable [3]. Until recently, relatively little attention has been paid to the effect of root temperature on the chilling response of plant leaves. Suzuki et al. [4,5] stated that root temperature is a very important factor for the response and susceptibility of leaves to chilling stress. In *Oryza sativa* visible damage to leaves exposed to light was observed when only leaves, but not roots, were chilled, whereas no visible damage was observed when both leaves and roots were chilled simultaneously [4]. Additionally, when only leaves, but not roots, were chilled PSII was photoinhibited, but not PSI [4]. These authors suggested that leaf chilling while root temperature is high blocks the electron transport between Q_A_ and Q_B_ in PSII, which causes the over-reduction of PSII in the light, although the biochemical basis of chilling stress has not been established [5].

Photosynthesis in chloroplasts involves a vectorial electron transfer from water in the lumen to NADP^+^ in the stroma, which is achieved by means of redox carriers. Besides this major pathway, based on functional measurements, alternative electron transfer pathways, involving non-photochemical reduction or the oxidation of plastoquinones at the expense of stromal electron donors or acceptors, have been proposed. These additional reactions cover two main concepts, one based on the cycling of electrons around PSI [6–8] and the other on chlororespiration [9,10], which consists of electron transfer reactions from stromal reductants to O_2_ through the plastoquinone pool [7,11,12]. Two thylakoidal enzymes, both of which are important in chlororespiration, have been molecularly characterised: the plastid-encoded NADH dehydrogenase (NDH) complex [13–18] and the nucleus-encoded plastid-localised terminal oxidase (PTOX) [19–21]. The NDH complex is an entry point for electrons into the photosynthetic electron-transport chain, involving the non-photochemical reduction of plastoquinones, and PTOX is a point of electron transfer from plastoquinol to molecular oxygen, resulting in the formation of water in the stroma and reducing the formation of reactive oxygen species (ROS) [11]. In addition to chlororespiration, the NDH complex is involved in the cyclic electron flow around PS I [22]. Two parallel cyclic pathways exist around PSI [22], one involving the NDH complex and the other sensitive to antimycin A, in which two proteins are essential components: the thylakoid membrane protein encoded by *pgr5* gene (PGR5) and the thylakoid transmembrane protein (PGRL1), which interacts functionally and physically with PGR5 [23–25]. The physiological roles of the chloroplast electron pathways operating around PSI have been difficult to establish. Although these reactions probably do not play a major role during photosynthesis under optimal conditions [26–29], they probably contribute to the flexibility of the electron transfer reactions required to balance ATP/NADPH requirements when photosynthesis operates under a changing environment [12,30–35].

Several studies have proposed that chlororespiratory components may be involved in the response mechanisms of plants to environmental stress, such as drought, heat and high light [19,36–50]. However, the possible role of chlororespiratory components in conditions of cold stress remain unclear [12]. It was reported that PTOX levels increased in alpine plant species acclimated to high light and low temperature [44,51] and in cold-acclimated *Arabidopsis thaliana* plants [52]. In contrast, no increase in the NDH complex under cold stress was observed [52]. Recently, we described the involvement of the chlororespiratory pathway, together with another pathway involving PGR5 polypeptide, in the tolerance to high light intensity under chilling stress of the cold-acclimated *Spathiphyllum wallisii*, a shade plant [53]. However, the extent of any cooperation between these pathways remains unclear and needs further investigation.

The present work studies the effects of cold on photosynthesis, as well as the involvement in the chilling stress of chlororespiratory enzymes and ferredoxin-mediated cyclic electron flow, in illuminated plants of *Hibiscus rosa-sinensis*, an ornamental of tropical origin with growth temperatures of 15–25°C.

## Materials and Methods

### Plant material and treatments

*Hibiscus rosa-sinensis* plants were grown in soil in 500 mL pots at 22–25°C in a greenhouse under natural light conditions and controlled watering to avoid drought stress until flowering. For the cold treatments, adult plants were transferred to cultivation chambers, where they were exposed to two 18 h photoperiods (500 μmol·m^-2^·s^-1^ PPFD supplied by white light lamps of 100 W Flood OSRAM, Augsburg, Germany) and different temperature treatments. Each 18 h photoperiod was followed by a 6 h night-period. The temperature treatments were as follows: 24°C/24°C (control conditions); 10°C/10°C and 10°C/24°C, representing stem/root temperatures, respectively. To change the temperature of the root with respect to the stem, a thermostatic circulator (LKB, GmbH, Germany) with a coil around the pot was used. The soil temperature was measured with a digital stem thermometer (Herter Instruments, Barcelona, Spain). In each treatment, the photoperiod temperature was the same as during the night-period. The measurements were made after the second night-period. The experiments were replicated in four separate plants for each treatment.

### Isolation of thylakoid membranes

Chloroplasts were isolated from leaves as described by Quiles and Cuello [13] using an extraction buffer (pH 7.6) containing 0.35 M sucrose, 25 mM Na-Hepes, 2 mM Na_2_-EDTA, 2 mM ascorbic acid, 4 mM dithiothreitol, 10 mM MgCl_2_ and 1 mM phenylmethylsulfonyl fluoride. As reported previously, a comparison of cytochrome c oxidase specific activity and the polypeptide profiles in mitochondrial and chloroplast fractions indicated that the chloroplast preparation was essentially mitochondrion-free [54]. The chloroplasts were washed twice and osmotically broken with 10 mM Tricine, 10 mM NaCl and 10 mM MgCl_2_ (pH 7.8) buffer as described previously [45]. The thylakoid membrane pellet was resuspended in a buffer (pH 7.5) containing 200 mM sorbitol, 130 mM KCl and 5 mM potassium phosphate at a chlorophyll concentration of 0.4 mg·mL^-1^, thus providing the suspension of the thylakoid membranes.

### Chlorophyll fluorescence measurements

Chlorophyll fluorescence was measured in the thylakoid membrane suspension (50 μg Chl mL^-1^) using a PAM-210 chlorophyll fluorometer (Heinz Walz GmbH, Effeltrich, Germany) and chlorophyll fluorescence was imaged, using the MINI-version of the Imaging-PAM (Heinz Walz GmbH, Effeltrich, Germany) in entire leaves before dawn, at the same temperature in the cultivation chambers as applied in the treatments. Prior to the fluorescence measurements, the samples were dark-adapted, the thylakoid membrane suspensions for 30 min and the entire leaves for 6h (night-period). First F_0_, the minimal fluorescence yield, and Fm, maximal fluorescence yield, were measured in dark-adapted samples. F_0_ was measured at a low frequency of pulse modulated measuring light, while Fm was measured with the help of a saturation pulse. This was followed by exposure of the entire leaves to actinic light (200 μmol·m^-2^·s^-1^ PPFD), measuring the fluorescence yield (F) and the maximal fluorescence yield in illuminated samples (F’_m_). The maximal quantum yield of PS II (F_v_/F_m_ = (F_m_-F_0_)/F_m_), the effective PS II quantum yield of illuminated samples (Y(II) = (Fm’-F)/Fm’), non-photochemical quenching (NPQ = (F_m_- F_m_’)/F_m_’)) and minimal fluorescence yield of illuminated sample (F_0_’ = F_0_/ (F_v_/F_m_ + F_0_/F_m_’)) were automatically calculated by the ImagingWin software (Heinz Walz GmbH, Effeltrich, Germany). Then light response curves were made in the entire leaves using pre-programmed rapid light curves of the PAM fluorometer software (Heinz Walz GmbH, Effeltrich, Germany) by illuminating the entire leaves with actinic light of different intensities (60, 90, 120, 150, 210, 310, 440, 600, 850 and 1250 μmol m^-2^ s^-1^ PAR), with 2 min illumination periods at each intensity. After each illumination period a saturation pulse was applied to determine the quantum yield of PS II and the relative electron transport rate, both of which were calculated using the PAM fluorometer software (Heinz Walz GmbH, Effeltrich, Germany).

### NADH dehydrogenase activity measurements

The NADH dehydrogenase activity of thylakoid membranes was determined by measuring NADH oxidation at 340 nm in a Perkin Elmer (Germany) spectrophotometer at 25°C, using decylplastoquinone (Sigma, USA) as electron acceptor. the NADH-plastoquinone oxidoreductase (NADH-PQR) activity was determined as described by Gamboa et al. [55]. One unit (U) of enzymatic activity is defined as the amount of enzyme preparation, which oxidized 1 μmol of substrate (NADH) per minute in the reaction conditions. The extinction coefficient of 6.22 mM^-1^·cm^-1^ at 340 nm was used to calculate the NADH oxidation rate.

### Gel electrophoresis and immunoblot analysis

SDS-PAGE was carried out in a linear gradient of 10–20% polyacrylamide gel (2.5% bis-acrylamide), as previously described [13]. Protein samples were denatured by the addition of 3.5% (w/v) SDS and 5% (v/v) mercaptoethanol and heating for 10 min at 70–80°C before being subjected to SDS-PAGE. Prestained SDS-PAGE standards (Bio-Rad Laboratories, USA) were used for immunoblot analyses. After SDS-PAGE, the polypeptides were electroblotted from gels onto polyvinylidene difluoride membranes (Immobilon-P, Millipore, USA). Transfer and immuno-detection of the blotted protein was carried out as described previously [13]. The secondary antibody was conjugated to alkaline phosphatase (Sigma, USA). Controls in which the primary and/or secondary antibodies were omitted during incubation did not reveal any bands.

### Other determinations

Protein was quantified using the method of Lowry et al. [56] after precipitation with 10% (w/v) trichloroacetic acid. Chlorophyll was determined by Lichtenthaler and Wellburn´s [57] method using 80% (v/v) acetone as solvent. Densitometric analysis and estimation of the polypeptide molecular masses were performed by ACTIB 1D digital image analyzer (Microptic, Barcelona, Spain). Analysis of variance (ANOVA) was used to compare the control and cold treatments.

## Results

### Photosynthetic parameters

**Fig 1:**
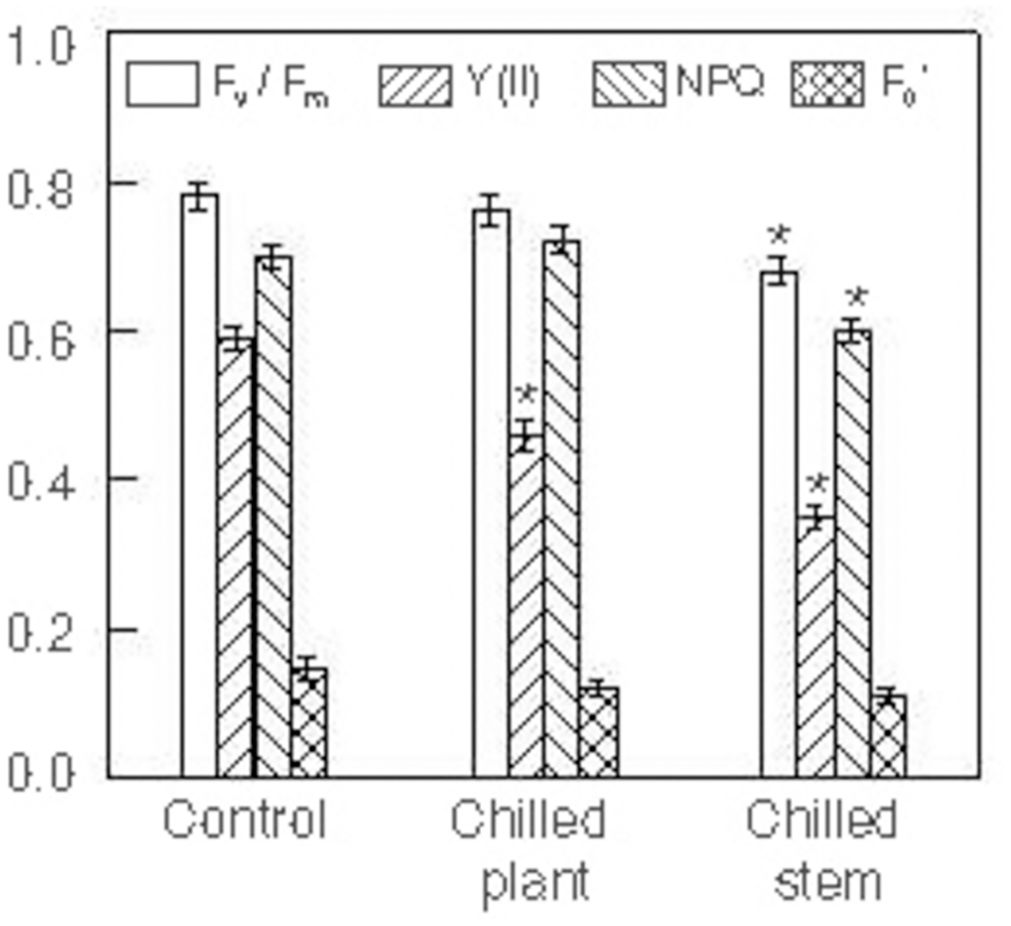
Photosynthetic parameters. Maximal quantum yield of PS II (Fv/Fm), effective PS II quantum yield (Y(II)), non-photochemical quenching (NPQ) and minimal fluorescence yield of illuminated sample (F_0_’) in intact leaves from *Hibiscus rosa-sinensis* plants after exposure to two photoperiods (18 h, 500 μmol·m^-2^·s^-1^ PPFD), with different temperatures maintained in the stem and in the root (°C stem/°C root): 24°C/24°C (control); 10°C/10°C (chilled plant) and 10°C/24°C (chilled stem). Each photoperiod was followed by a 6 h night-period, maintaining the same temperatures as in the photoperiod. The values are means ± SE from four separate plants for each treatment, with duplicate measurements in each plant. Significant differences are indicated by an asterisk (ANOVA, *p*<0.05).

The fluorescence imaging technique was used to test photosynthesis in intact leaves from plants exposed to two photoperiods with different temperatures in the stem and root (°C stem/°C root): 24°C/24°C (control), 10°C/10°C (chilled plant) and 10°C/24°C (chilled stem). Fig 1 shows the values of the maximal quantum yield of PS II (F_v_/F_m_), the effective PS II quantum yield (Y(II)), the non-photochemical quenching (NPQ) and minimal fluorescence yield of illuminated sample (F_0_’). The F_v_/F_m_ values in plants exposed to cold were lower than the values of control plants, although a significant difference was only observed in plants exposed to 10°C/24°C. The effective PSII quantum yield decreased significantly in the plants exposed to both cold treatments, the lowest values being seen in 10°C/24°C plants. The NPQ, which is an indicator of thermal dissipation [58], did not increase significantly in plants exposed to 10°C/10°C, but decreased significantly in plants exposed to 10°C/24°C.

**Fig 2:**
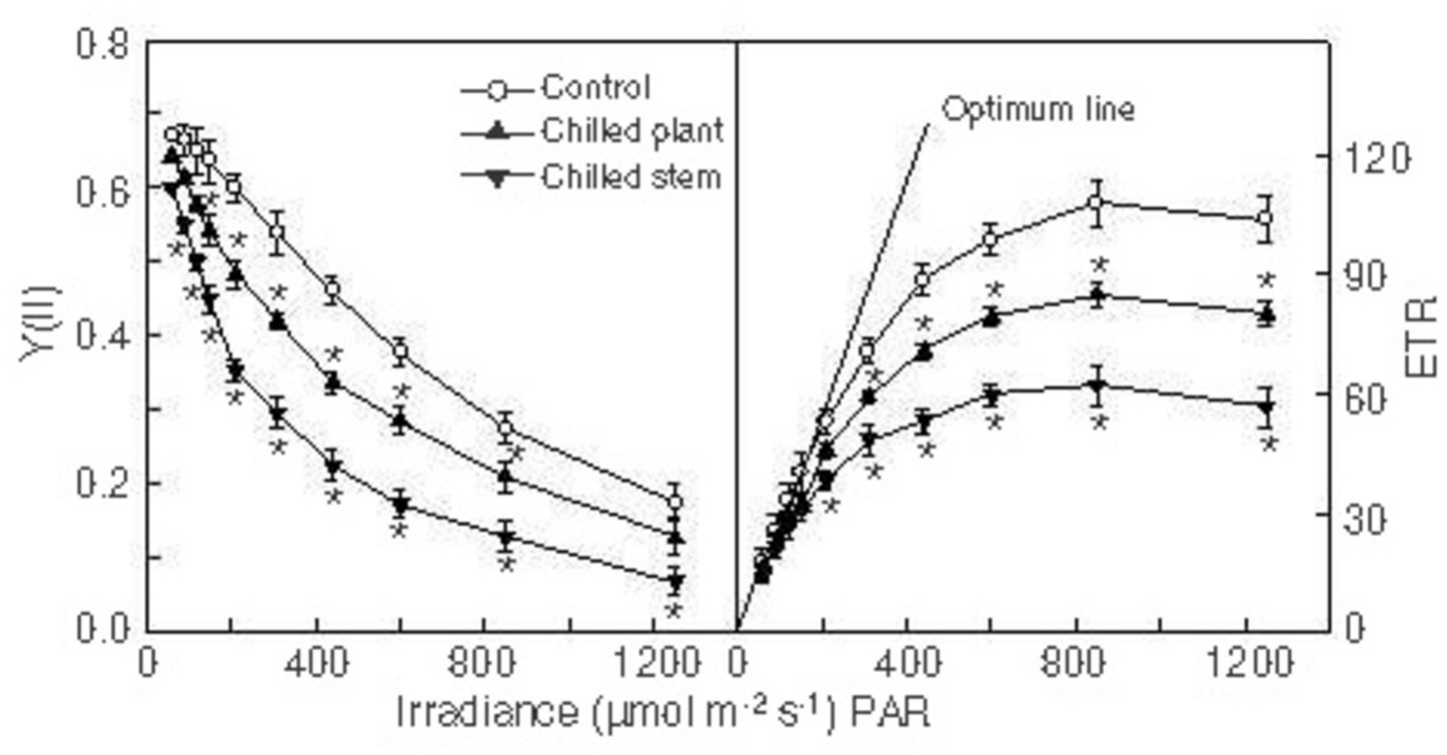
Light response curves. Light response curves for the quantum yield of PS II (Y(II)) and the relative electron transport rate (ETR) in intact leaves from *Hibiscus rosa-sinensis* plants after exposure to two photoperiods (18 h, 500 μmol·m^-2^·s^-1^ PPFD), with different temperatures maintained in the stem and in the root (°C stem/°C root): 24°C/24°C (control); 10°C/10°C (chilled plant) and 10°C/24°C (chilled stem). Each photoperiod was followed by a 6 h night-period, maintaining the same temperatures as in the photoperiod. The values are means ± SE from four separate plants for each treatment, with duplicate measurements in each plant. Significant differences are indicated by an asterisk (ANOVA, *p*<0.05).

The light response curves for the quantum yield of PSII and the relative electron transport rate (ETR) in intact leaves from plants in control conditions and those exposed to the cold treatments are shown in Fig 2. The quantum yield of PSII decreased with increasing photonic flux density in all cases, because electrons accumulated on the PSII acceptor side and there was a relative increase in non-radiative energy dissipation. When light was not excessive, the PSII quantum yield maintained its maximum value and the relationship between the relative electron transport rate and light intensity was linear (optimum line). The relative electron transport rate fell below the values predicted by the optimum line when the PSII quantum yield decreased because the light became excessive. Eventually a saturated rate was reached, which represents the photosynthetic electron transport capacity. This capacity, as well as the PSII quantum yields and the relative electron transport rates, were lower in plants exposed to cold temperatures than in control plants, although the differences with respect to the control values were greater in plants exposed to 10°C/24°C (chilled stem) than in those exposed to 10°C/10°C (chilled plant).

### NADH-PQR activity and electron donation to plastoquinone in the thylakoid membranes

**Fig 3:**
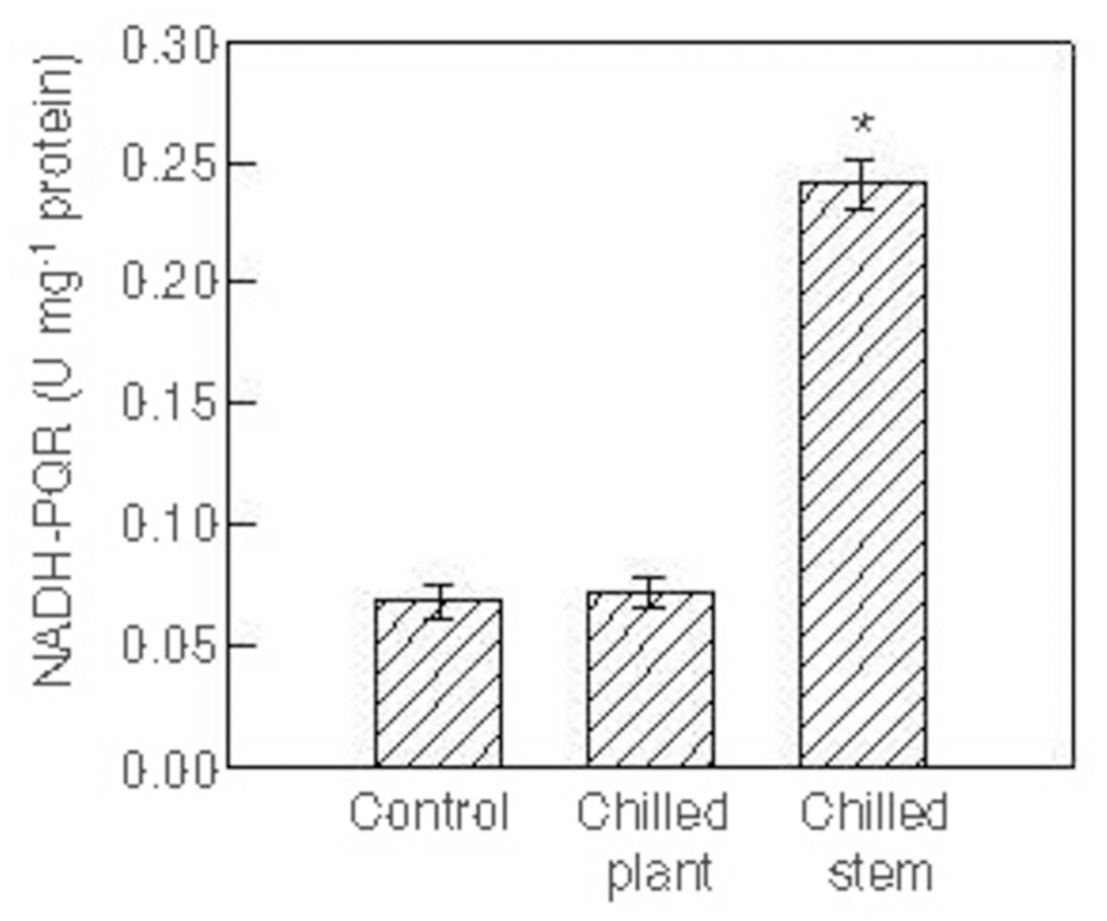
NADH-plastoquinone oxidoreductase activity. NADH-plastoquinone oxidoreductase (NADH-PQR) activity in the thylakoid membranes isolated from leaves of *Hibiscus rosa-sinensis* plants after exposure to two photoperiods (18 h, 500 μmol·m^-2^·s^-1^ PPFD), with different temperatures maintained in the stem and in the root (°C stem/°C root): 24°C/24°C (control); 10°C/10°C (chilled plant) and 10°C/24°C (chilled stem). Each photoperiod was followed by a 6 h night-period, maintaining the same temperatures as in the photoperiod. The values are means ± SE from four separate plants for each treatment, with duplicate measurements in each plant. Significant differences are indicated by an asterisk (ANOVA, *p*<0.05).

The NDH activity was assayed as NADH-plastoquinone oxidoreductase (NADH-PQR) activity in thylakoid membranes isolated from leaves of plants exposed to two photoperiods with different temperatures (Fig 3). The activity was similar in plants exposed to 24°C/24°C (control) and 10°C/10°C (chilled plant). However, in plants exposed to 10°C/24°C (chilled stem) the activity was approximately 3.5 times higher than in the control.

**Fig 4:**
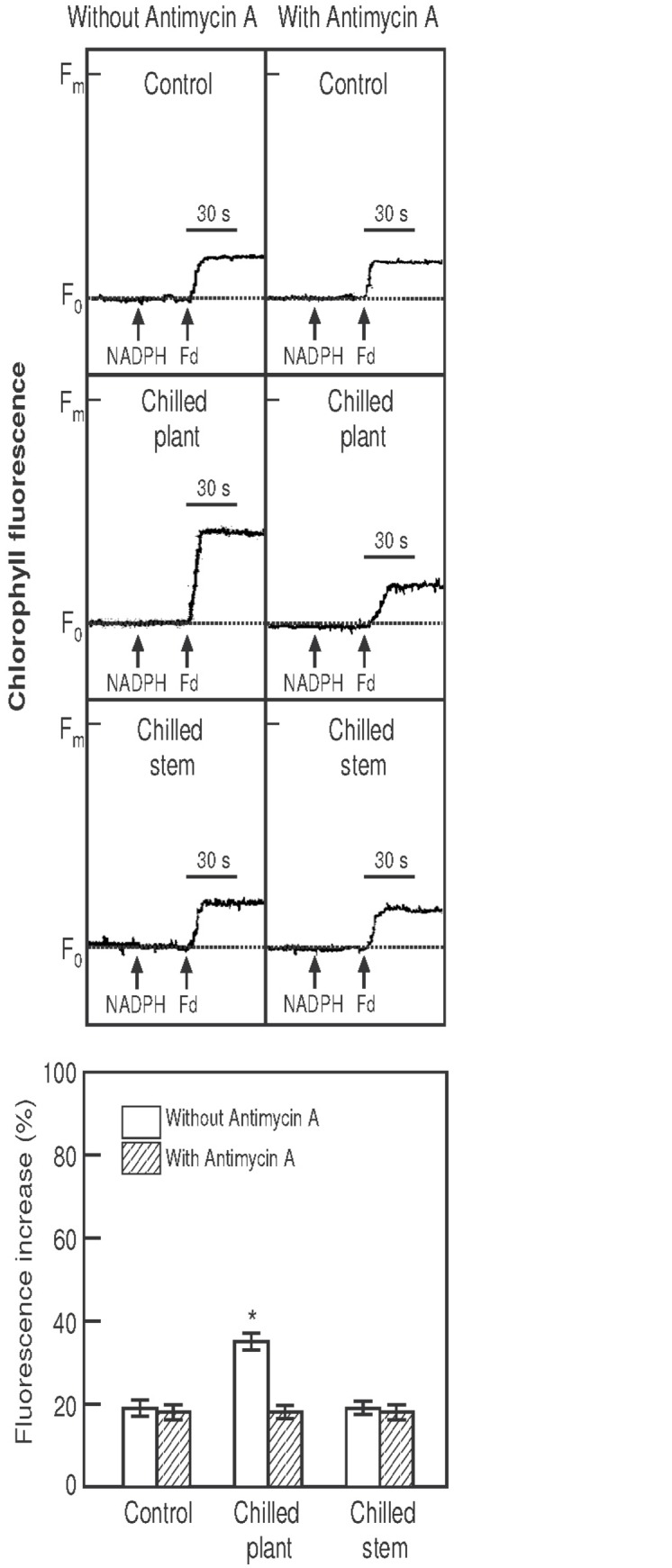
Fluorescence increase. Increases in chlorophyll fluorescence after the addition of NADPH (0.25 mM) 30 s into the run and ferredoxin (5 μM) 60 s into the run, under weak measuring light (1.0 μmol·m^-2^·s^-1^) in thylakoid membranes (50 μg Chl·mL^-1^) isolated from recently detached leaves of *Hibiscus rosa-sinensis* plants after exposure to two photoperiods (18 h, 500 μmol·m^-2^·s^-1^ PPFD), with different temperatures maintained in the stem and in the root (°C stem/°C root): 24°C/24°C (control); 10°C/10°C (chilled plant) and 10°C/24°C (chilled stem). Each photoperiod was followed by a 6 h night-period, maintaining the same temperatures as in the photoperiod. Additionally, thylakoid suspensions were incubated with antimycin A (2 μM) for 2 min prior to the measurements. The figure shows typical curves and the histograms of the means ± SE from four separate plants for each treatment, with duplicate measurements in each plant. Significant differences are indicated by an asterisk (ANOVA, *p*<0.05).

The activity of electron donation by NADPH and ferredoxin to plastoquinone was assayed as an increase in the chlorophyll fluorescence emitted during exposure to light of a very low intensity (1.0 μmol m^-2^ s^-1^) [45], at which intensity, the fluorescence level predominantly reflects the reduction of plastoquinone by electron transport from ferredoxin [23]. Chlorophyll fluorescence increased after the addition of NADPH (0.25 mM) and ferredoxin (5 μM) under weak measuring light (Fig 4) in thylakoid membrane suspensions (50 μg Chl mL-1) isolated from the chloroplasts of leaves recently detached from plants exposed to two photoperiods with different temperatures. Additionally, thylakoid suspensions were incubated with antimycin A (2 μM) for 2 min prior to the measurements. Control plants and plants exposed to 10°C/24°C (chilled stem) showed a similar increase in chlorophyll fluorescence. However, plants exposed to 10°C/10°C (chilled plant) showed a more pronounced increase in chlorophyll fluorescence (approximately 2 times the control level). Antimycin A inhibited the increase in chlorophyll fluorescence compared with measurements made without inhibitor, although its effect was slight in the control and the 10°C/24°C plants.

The results suggest that cyclic electron transport was stimulated in leaves only when the whole plant was chilled, while chlororespiration was stimulated in leaves when the roots were not chilled and kept at 24°C and the stem was cooled to 10°C.

### Immunoblot analysis

**Fig 5:**
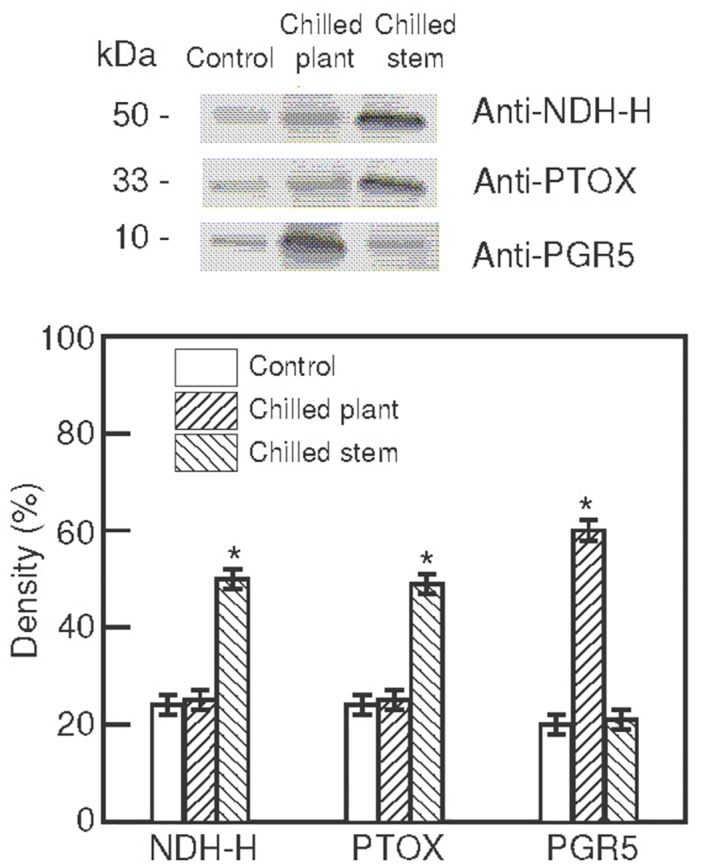
Immunoblot analysis. Immunoblot analysis of the thylakoid polypeptides isolated from leaves of *Hibiscus rosa-sinensis* plants after exposure to two photoperiods (18 h, 500 μmol·m^-2^·s^-1^ PPFD), with different temperatures maintained in the stem and in the root (°C stem/°C root): 24°C/24°C (control); 10°C/10°C (chilled plant) and 10°C/24°C (chilled stem). Each photoperiod was followed by a 6 h night-period, maintaining the same temperatures as in the photoperiod. Twenty micrograms per well of the thylakoid proteins were separated by SDS-PAGE prior to immunoblot assays and tested with anti-NDH-H, anti-PTOX, and anti-PGR5 antibodies. The molecular masses of the polypeptides are indicated in the margin. The immunoblots from three independent experiments were analysed by video densitometric analysis. In each case, the total density of the samples (control + chilled plant + chilled stem) was taken as 100%. The values are means ± SE. Significant differences are indicated by an asterisk (ANOVA, *p*<0.05).

Thylakoid membranes isolated from leaves of plants exposed to two photoperiods with different temperatures were used for immunoblot analyses, using specific antibodies against the NDH-H subunit of the thylakoidal NDH complex (anti-NDH-H), PTOX and PGR5. Fig 5 shows that the antibody anti-NDH-H clearly recognised only one polypeptide of 50 kDa, while the antibodies anti-PTOX and anti-PGR5 recognised others of 33 kDa and 10 kDa, respectively, in each thylakoid membrane sample. No additional polypeptide was recognised by the antibodies assayed (not shown). Immunoblots were analysed densitometrically and the results are also shown in Fig 5. The levels of PTOX and the NDH-H subunit of the NDH complex were higher in the thylakoids of plants exposed to 10°C/24°C (chilled stem) than in leaves from control plants. However, the levels in leaves from plants exposed to 10°C/10°C (chilled plant) remained similar to the control. In contrast, the PGR5 polypeptide levels only increased in plants exposed to 10°C/10°C and remained similar to the control in leaves from plants exposed to 10°C/24°C.

## Discussion

The tropical species *Hibiscus rosa-sinensis* is sensitive to chilling stress. Under cold conditions PSII was inhibited and photosynthetic linear electron transport was down-regulated, as indicated by a reduction in the photochemistry efficiency of PSII and in the electron transport capacity. However, the susceptibility of leaves to cold may be modified by the root temperature, we have seen in this work that when the roots were kept at 24°C while the stem was cooled to 10°C, the quantum yield of PSII and the relative electron transport rates were much lower than when the whole plant (root and stem) was exposed to 10°C. In rice seedlings Suzuki et al. [4,5] also observed that PSII activity was severely inhibited and that NPQ and photosynthetic electron transport decreased when shoots, but not roots, were cooled. These authors suggested [5] that the chilling of rice leaves accompanied by high root temperature blocked the electron transport between Q_A_ and Q_B_ in PSII, leading to the over-reduction of PSII in the light, the blockage being attributed to an interaction of a certain molecule with a component or components of PSII. Suzuki et al. [5] also suggested that this molecule was probably nitric oxide, which to have the potential to bind to the Q_A_-Fe^2+^-Q_B_ complex, thereby inhibiting electron transfer from Q_A_^-^ to Q_B_. Nitric oxide may be produced when nitrate is accumulated and, more recently, Suzuki et al. [59] described that the malfunction of photosynthesis induced by high root temperature is associated with the accumulation of nitrate and nitrite in leaves.

The inhibition of photosynthetic linear electron transport, the main electron transfer pathway, could trigger alternative pathways, such as cyclic electron flow around PSI and chlororespiration [12], since regulation of the flow of photosynthetic electrons is essential for the health and survival of plants. These alternative ways of photosynthetic linear electron transport may contribute to protecting the photosynthetic apparatus under stress conditions [12,24,35,43,45,46,48,52,55,60–62]. Recently, we described the involvement of the chlororespiratory pathway in the tolerance to high light intensity under chilling stress [53]. However, the extent of any cooperation between chlororespiration and alternative photosynthetic electron transport pathways, such as cyclic electron flow around PSI involving PGR5 polypeptide, was unclear [53]. In the present work, it was observed that the activity of electron donation by NADPH and ferredoxin to plastoquinone, which was inhibited by antimycin A, increased in relation to the control, only when the whole plant, root and stem, was exposed to 10°C. Additionally, the levels of PGR5 polypeptide, an essential component of cyclic electron flow sensitive to antimycin A [23], also increased in plants where both roots and stems were exposed to 10°C, suggesting that cyclic electron transport is stimulated only when the whole plant is chilled. However, temperatures of 10°C/24°C (stem/root) did not increase the activity of electron donation by NADPH and ferredoxin to plastoquinone, probably because, at these temperatures, a malfunction in electron transport somewhere between PSII and PSI there was, as suggested by Suzuki et al. [5]. This malfunction blocks both linear and cyclic electron flows, leading to the over-reduction of PSII in the light [5]. Interestingly, both NDH complex and PTOX increased only in plants exposed to 10°C/24°C (stem/root), suggesting that chororespiration was stimulated only when the roots were kept at 24°C and the stem was cooled to 10°C.

The concerted action of NDH complex and PTOX would optimize the efficiency of the cyclic pathways, preventing over-reduction of the electron transfer chain [12,63] and reducing the accumulation of reactive oxygen species by recycling electrons to the plastoquinone pool and, ultimately, to oxygen through PTOX, forming water in the stroma. Additionally, these pathways contribute to balancing ATP/NADPH requirements and to generating a large proton gradient and acidification of the lumen, which play a crucial role in the regulation, preventing the light-induced inactivation of both PSI and PSII through the formation of non-photochemical quenching [12,35].

In conclusion, this work has shown that illuminated plants were more tolerant to chilling when both stem and roots were chilled. In such conditions, neither NDH complex nor PTOX increased. However, both the activity of electron donation by NADPH and ferredoxin to plastoquinone and the amount of PGR5 polypeptide, an essential component of cyclic electron flow around PSI, increased, suggesting that in these conditions cyclic electron flow helps protect photosystems. However, when the stems but not the roots were cooled, cyclic electron flow did not increase and the efficiency of PSII photochemistry decreased considerably as a result of insufficient dissipation of the excess light energy. In such conditions, both chlororespiratory enzymes, NDH complex and PTOX, increased. The apparent correlation between the low functioning of PSII and the up-regulation of PTOX and the thylakoidal NDH complex supports a role for chlororespiration in the response of plants to chilling stress when other pathways, such as cyclic electron flow around PSI, are insufficient to protect PSII.

## References

[1] Lyons JM (1973) Chilling injury in plants. Annu. Rev. Plant Physiol. 24: 445–466.

[2] Allen DJ, Ort DR (2001) Impacts of chilling temperatures on photosynthesis in warm-climate plants. Trends Plant Sci. 9: 36–42. 10.1016/s1360-1385(00)01808-2 11164376

[3] Sonoike K (1998) Photoinhibition of photosystem I in chilling sensitive plants determined in vivo and in vitro, in: Garab G. (Ed.), Photosynthesis: Mechanisms and Effects. Kluwer Academic Publishers, Dordrecht, The Netherlands, pp. 2217–2220.

[4] Suzuki K, Nagasuga K, Okada M (2008) The chilling injury induced by high root temperature in the leaves of rice seedlings. Plant Cell Physiol. 49: 433–442. 10.1093/pcp/pcn020 18252732

[5] Suzuki K, Ohmori Y, Ratel E (2011) High root temperature blocks both linear and cyclic electron transport in the dark during chilling of the leaves of rice seedlings. Plant Cell Physiol. 52: 1697–1707. 10.1093/pcp/pcr104 21803813

[6] Fork DC, Herbert SK (1993) Electron transport and photophosphorylation by Photosystem I *in vivo* in plants and cyanobacteria. Photosynth. Res. 36: 149–168. 10.1007/BF00033035 24318920

[7] Bukhov N, Carpentier R (2004) Alternative photosystem I-driven electron transport routes: mechanisms and functions. Photosynth. Res. 82: 17–33. 16228610 10.1023/B:PRES.0000040442.59311.72

[8] Johnson GN (2005) Cyclic electron transport in C3 plants: fact or artefact? J. Exp. Bot. 56: 407–416. 15647314 10.1093/jxb/eri106

[9] Bennoun P (1982) Evidence for a respiratory chain in the chloroplast. Proc. Natl. Acad. Sci. U.S.A. 79: 4352–4356. 16593210 10.1073/pnas.79.14.4352 PMC346669

[10] Bennoun P (1994) Chororespiration revised: mitochondrial-plastid interaction in Chlamydomonas. Bioch. Biophys. Acta 1186: 59–66.

[11] Peltier G, Cournac L (2002) Chlororespiration. Annu. Rev. Plant Biol. 53: 53–550. 10.1146/annurev.arplant.53.100301.135242 12227339

[12] Rumeau D, Peltier G, Cournac L (2007) Chlororespiration and cyclic electron flow around PSI during photosynthesis and plant stress response. Plant Cell Environ. 30: 1041–1051. 17661746 10.1111/j.1365-3040.2007.01675.x

[13] Quiles MJ, Cuello J (1998) Association of ferredoxin-NADP oxidoreductase with the chloroplastic pyridine nucleotide dehydrogenase complex in barley leaves. Plant Physiol. 117: 235–244. 9576793 10.1104/pp.117.1.235 PMC35008

[14] Sazanov LA, Burrows PA, Nixon PJ (1998) The plastid *ndh* genes code for an NADH-specific dehydrogenase: Isolation of a complex I analogue from pea thylakoid membranes. Proc. Natl. Acad. Sci. U.S.A. 95: 1319–1324. 9448329 10.1073/pnas.95.3.1319 PMC18756

[15] Quiles MJ, García A, Cuello J (2000) Separation by blue-native PAGE and identification of the whole NAD(P)H dehydrogenase complex from barley stroma thylakoids. Plant Physiol. Biochem. 38: 225–232.

[16] Quiles MJ (2005) Regulation of the expression of chloroplast *ndh* genes by light intensity applied during oat plant growth. Plant Sci. 168: 1561–1569.

[17] Rumeau D, Becuwe-Linka N, Beyly A, Louwagie M, Garin J, Peltier G (2005) New subunits NDH-M, -N, and –O, encoded by nuclear genes, are essential for plastid Ndh complex functioning in higher plants. Plant Cell 17: 219–232. 15608332 10.1105/tpc.104.028282 PMC544500

[18] Ifuku K, Endo T, Shikanai T, Aro EM (2011) Structure of the Chloroplast NADH Dehydrogenase-Like Complex: Nomenclature for Nuclear-Encoded Subunits. Plant Cell Physiol. 52: 1560–1568. 10.1093/pcp/pcr098 21785130

[19] Aluru MR, Rodermel SR (2004) Control of chloroplast redox by the IMMUTANS terminal oxidase. Physiol. Plant. 120: 4–11. 15032871 10.1111/j.0031-9317.2004.0217.x

[20] Kuntz M (2004) Plastid terminal oxidase and its biological significance. Planta 218: 896–899. 14986142 10.1007/s00425-004-1217-6

[21] Sun X, Wen T (2011) Physiological roles of plastid terminal oxidase in plant stress responses. J. Biosci. 36: 951–956. 22116293 10.1007/s12038-011-9161-7

[22] Joët T, Cournac L, Horvath EM, Medgyesy P, Peltier G (2001) Increased sensitivity of photosynthesis to antimycin A induced by inactivation of the chloroplast *ndhB* gene. Evidence for a participation of the NADH-dehydrogenase complex to cyclic electron flow around photosystem I. Plant Physiol. 125: 919–1929. 10.1104/pp.125.4.1919 PMC88847 11299371

[23] Munekage Y, Hojo M, Meurer J, Endo T, Tasaka M, Shikanai T (2002) PGR5 is involved in cyclic electron flow around photosystemI and is essential for photoprotection in Arabidopsis. Cell 110: 361–371. 12176323 10.1016/s0092-8674(02)00867-x

[24] Munekage Y, Hashimoto M, Miyake C, Tomizawa K, Endo T, Tasaka M, et al (2004) Cyclic electron flow around photosystem I is essential for photosynthesis. Nature 429: 579–582. 15175756 10.1038/nature02598

[25] DalCorso G, Pesaresi P, Masiero S, Aseeva E, Schünemann D, Finazzi G, et al (2008) A complex containing PGRL1 and PGR5 is involved in the switch between linear and cyclic electron flow in *Arabidopsis* . Cell 132: 273–285. 10.1016/j.cell.2007.12.028 18243102

[26] Hebert SK, Fork DC, Malkin S (1990) Photoacoustihc measurements in vivo of energy storage by cyclic electron flow in algae and higher plants. Plant Physiol. 94: 926–934. 16667873 10.1104/pp.94.3.926 PMC1077324

[27] Havaux M, Greppin H, Strasser RJ (1991) Functioning of photosystem I and photosystem II in pea leaves exposed to heat stress in the presence of absence of light, analysis using in vivo fluorescence, absorbency, oxygen and photoacoustic measurements. Planta 186: 88–98. 10.1007/BF00201502 24186579

[28] Bendall DS, Manasse RS (1995) Cyclic photophosphorylation and electron transport. Biochim. Biophys. Acta 1229, 23–38.

[29] Joët T, Cournac L, Peltier G, Havaux M (2002) Cyclic electron flow around photosystem I in C3 plants. In vivo control by the redox state of chloroplasts and involvement of the NADH- dehydrogenase complex. Plant Physiol. 128: 760–769. 11842179 10.1104/pp.010775 PMC148937

[30] Joliot P, Joliot A (2002) Cyclic electron transfer in plant leaf. Proc. Natl. Acad. Sci. U.S.A. 99: 10209–10214. 12119384 10.1073/pnas.102306999 PMC126649

[31] Joliot P, Joliot A (2006) Cyclic electron flow in C3 plants. Biochim. Biophys. Acta 1757: 362–368. 16762315 10.1016/j.bbabio.2006.02.018

[32] Golding AJ, Johnson GN (2003) Down-regulation of linear and activation of cyclic electron transport during drought. Planta 218: 107–114. 12883889 10.1007/s00425-003-1077-5

[33] Golding AJ, Finazzi G, Jhonson GN (2004) Reduction of the thylakoid electron transport chain by stromal reductants, evidence for activation of cyclic electron transport upon dark adaptation or under drought. Planta 220: 356–363. 15316779 10.1007/s00425-004-1345-z

[34] Breyton C, Nandha B, Johnson GN, Joliot P, Finazzi G (2006) Redox modulation of cyclic electron flow around photosystem I in C3 plants. Biochemistry 45: 13465–13475. 17087500 10.1021/bi061439s

[35] Joliot P, Johnson GN (2011) Regulation of cyclic and linear electron flow in higher plants. Proc. Natl. Acad. Sci. U.S.A. 108: 13317–13322. 10.1073/pnas.1110189108 21784980 PMC3156182

[36] Herbert SK, Martin RE, Fork DC (1995) Light adaptation of cyclic electron transport through photosystem I in the cyanobacterium *Synechococcus* sp PCC 7942. Photosynth Res.46: 277–285. 10.1007/BF00020441 24301593

[37] Martín M, Casano LM, Sabater B (1996) Identification of the product of *ndh* A gene as a thylakoid protein synthesized in response to photooxidative treatment. Plant Cell Physiol. 37: 293–298. 8673340 10.1093/oxfordjournals.pcp.a028945

[38] Catalá R, Sabater B, Guera A (1997) Expression of the plastid *ndh*F gene product in photosynthetic and non-photosynthetic tissues of developing barley seedlings. Plant Cell Physiol. 38: 1382–1388. 9522468 10.1093/oxfordjournals.pcp.a029133

[39] Endo T, Shikanai T, Takabayashi A, Asada K, Mi H, Sato F (1999) The role of chloroplastic NAD(P)H dehydrogenase in photoprotection. FEBS Lett. 457: 5–8. 10486552 10.1016/s0014-5793(99)00989-8

[40] Teicher HB, Moller BL, Scheller HV (2000) Photoinhibition of photosystem I in field-grown barley (*Hordeum vulgare* L.): Induction, recovery and acclimation. Photosynth. Res. 64: 53–61. 16228443 10.1023/A:1026524302191

[41] Casano LM, Martín M, Sabater B (2001) Hydrogen peroxide mediates the induction of chloroplastic Ndh complex under photooxidative stress in barley. Plant Physiol. 125: 1450–1458. 11244124 10.1104/pp.125.3.1450 PMC65623

[42] Rizhsky L, Hallak-Herr E, van Breusegem F, Rachmilevitch S, Barr JE, Rodermel S, et al (2002) Double antisense plants lacking ascorbate peroxidase and catalase are less sensitive to oxidative stress than single antisense plants lacking ascorbate peroxidase or catalase. Plant J. 32: 329–342. 12410811 10.1046/j.1365-313x.2002.01427.x

[43] Quiles MJ, López NI (2004) Photoinhibition of photosystems I and II induced by exposure to high light intensity during oat plant growth. Effects on the chloroplast NADH dehydrogenase complex. Plant Sci. 166: 815–823.

[44] Streb P, Josse EM, Gallouët E, Baptist F, Kuntz M, Cornic G (2005) Evidence for alternative electron sinks to photosynthetic carbon assimilation in the high mountain plant species *Ranunculus glacialis* . Plant Cell Environ. 28: 1123–113.

[45] Quiles MJ (2006) Stimulation of chlororespiration by heat and high light intensity in oat plants. Plant Cell Environ. 29: 1463–1470. 16898010 10.1111/j.1365-3040.2006.01510.x

[46] Díaz M, De Haro V, Muñoz R, Quiles MJ (2007) Chlororespiration is involved in the adaptation of *Brassica* plants to heat and high light intensity. Plant Cell Environ. 30: 1578–1585. 17944817 10.1111/j.1365-3040.2007.01735.x

[47] Tallón C, Quiles MJ (2007) Acclimation to heat and high light intensity during the development of oat leaves increases the NADH DH complex and PTOX levels in chloroplasts. Plant Sci. 173: 438–445.

[48] Ibañez H, Ballester A, Muñoz R, Quiles MJ (2010) Chlororespiration and tolerance to drought, heat and high illumination. J. Plant Physiol. 167: 732–738. 10.1016/j.jplph.2009.12.013 20172620

[49] Paredes M, Quiles MJ (2013) Stimulation of chlororespiration by drought under heat and high illumination in *Rosa meillandina* . J. Plant Physiol. 170: 165–171. 10.1016/j.jplph.2012.09.010 23122789

[50] Muñoz R, Quiles MJ (2013) Water deficit and heat affect the tolerance to high illumination in Hibiscus plants. Int. J. Mol. Sci. 14: 5432–5444. 10.3390/ijms14035432 23470922 PMC3634501

[51] Laureau C, Bligny R, Streb P (2011) The significance of glutathione for photoprotection at contrasting temperatures in the alpine plant species *Soldanella alpine* and *Ranunculus glacialis* . Physiol. Plant. 143: 246–260. 10.1111/j.1399-3054.2011.01505.x 21848651

[52] Ivanov AG, Rosso D, Savitch LV, Stachula P, Rosembert M, Oquist G, et al (2012) Implications of alternative electron sinks in increased resistance of PSII and PSI photochemistry to high light stress in cold-acclimated *Arabidopsis thaliana* . Photosynth. Res. 113: 191–206. 10.1007/s11120-012-9769-y 22843101

[53] Segura MV, Quiles MJ (2015) Involvement of chlororespiration in chilling stress in the tropical species Spathiphyllum wallisii. Plant Cell Environ. 38: 525–533. 10.1111/pce.12406 25041194

[54] Quiles MJ, García A, Cuello J (2003) Comparison of the thylakoidal NAD(P)H dehydrogenase complex and the mitochondrial complex I separated from barley by blue-native PAGE. Plant Sci. 164: 541–547.

[55] Gamboa J, Muñoz R, Quiles MJ (2009) Effects of antimycin A and n-propyl gallate on photosynthesis in sun and shade plants. Plant Sci. 177: 643–647.

[56] Lowry OH, Rosebrough NJ, Farr AL, Randall RJ (1951) Protein measurement with the Folin phenol reagent. J. Biol. Chem. 193: 265–275. 14907713

[57] Lichtenthaler HK, Wellburn AR (1983) Determinations of total carotenoids and chlorophylls a and b of leaf extracts in different solvents. Biochem. Soc. Trans. 11: 591–592.

[58] Demming-Adams B, Adams WWI (1996) Xanthophyll cycle and light stress in nature: uniform response to excess direct sunlight among higher plant species. Planta 198: 460–470

[59] Suzuki K, Ohmori Y, Nagao M (2013) Accumulation of Nitrate and Nitrite in chilled leaves of rice seedlings is induced by high root temperature. Plant Cell Physiol. 54: 1769–1779. 10.1093/pcp/pct120 23975886

[60] Li XG, Duan W, Meng QW, Zou Q, Zhao SJ (2004) The function of chloroplastic NAD(P)H dehydrogenase in tobacco during chilling stress under low irradiance. Plant Physiol. 45: 103–108. 10.1093/pcp/pch011 14749491

[61] Wang P, Duan W, Takabayashi A, Endo T, Shikanai T, Ye JY, et al (2006) Chloroplastic NAD(P)H dehydrogenase in tobacco leaves functions in alleviation of oxidative damage caused by temperature stress. Plant Physiol. 141: 465–474. 16428601 10.1104/pp.105.070490 PMC1475475

[62] Saura P, Quiles MJ (2009) Assessment of Photosynthesis Tolerance to Herbicides, Heat and High Illumination by Fluorescence Imaging. Open Plant Sci. J. 3: 7–13.

[63] Havaux M, Rumeau D, Ducruet JM (2005) Probing the FQR and NDH activities involved in cyclic electron transport around photosystem I by the ‘afterglow’ luminescence. Biochim. Biophys. Acta 1709: 203–213. 16137641 10.1016/j.bbabio.2005.07.010

